# Draft Genome Sequence of Neisseria mucosa Strain HSUH001, Isolated from an Aggressive Periodontal Lesion

**DOI:** 10.1128/MRA.00238-21

**Published:** 2021-05-13

**Authors:** Masatoshi Yamashita, Toshiyuki Nagasawa, Satsuki Kato, Hiroshi Miyakawa, Mari Fujita, Yasushi Furuichi, Keiji Nagano

**Affiliations:** a Division of Microbiology, Department of Oral Biology, School of Dentistry, Health Sciences University of Hokkaido, Hokkaido, Japan; b Division of Periodontology and Endodontology, Department of Oral Rehabilitation, School of Dentistry, Health Sciences University of Hokkaido, Hokkaido, Japan; University of Maryland School of Medicine

## Abstract

We report the draft genome sequence (143 contigs, with a total length of 2,424,805 bp and an *N*_50_ value of 36,066 bp) of a bacterium isolated from an aggressive periodontal lesion in a patient. We assigned strain HSUH001 to Neisseria mucosa through a multilocus sequence analysis.

## ANNOUNCEMENT

Periodontitis develops as a response to the colonization of multiple species of bacteria in subgingival tissues through biofilm formation (1). Aggressive periodontitis, which is a type of periodontal disease, progresses rapidly, despite limited biofilm formation. Although Aggregatibacter actinomycetemcomitans, a Gram-negative facultative anaerobe, is a representative pathogen associated with the disease, studies have indicated the involvement of other bacteria in exacerbation of the disease (2).

To isolate the major bacterial species from an aggressive periodontal lesion, we used a medium selective for A. actinomycetemcomitans, consisting of tryptic soy agar supplemented with 0.1% yeast extract, 10% inactivated horse serum, 75 μg/ml bacitracin, and 5 μg/ml vancomycin (3). This medium also allows other bacteria, including the *Neisseria* species, to grow (4). The dental biofilm sample was obtained from an aggressive periodontal lesion. This study was approved by the institutional review board of the Health Sciences University of Hokkaido, Research Ethics Committee (approval number 52). The sample was spread onto the agar medium and incubated at 37°C for several days in an incubator supplemented with 5% CO_2_. The predominant colony type was observed to be white, small, and smooth (5). One of the colonies was repeatedly streaked onto the agar medium for isolation and was stored at −80°C. We named this strain HSUH001. After cultivation of a single colony in brain heart infusion broth at 37°C for 12 h in an incubator supplemented with 5% CO_2_, the genomic DNA of HSUH001 was extracted using the Wizard genomic DNA purification kit (Promega Corp., Madison, WI, USA) and was subjected to genomic DNA sequencing. A sequence library was prepared with the HiSeq X Ten reagent kit v2.5 (FC-5001; Illumina, Inc., San Diego, CA, USA). Sequencing was conducted using a next-generation sequencer, the Illumina HiSeq X platform (150-bp paired-end reads). After the raw sequences (30,487,744 reads) were trimmed and quality filtered (Chastity filter; Illumina, Inc.), 828,782 reads remained, providing approximately 50-fold genome coverage of HSUH001. These sequence data were subjected to bioinformatic analysis, including genome assembly. Default parameters were used for all analyses. The reads were assembled *de novo* using Platanus_B v1.0 (http://platanus.bio.titech.ac.jp/platanus-b) (6). After removal of contigs shorter than 200 bp, the final draft assembly consisted of 143 contigs, with a total length of 2,424,805 bp, an *N_50_* value of 36,066 bp, and a G+C content of 51.4%. Prokka v1.14.5 (https://github.com/tseemann/prokka/releases) (7), a bacterial genome annotation program, predicted 2,133 protein-coding genes, 1 rRNA gene, 52 tRNA genes, and 2 clustered regularly interspaced short palindromic repeat (CRISPRs).

The best BLAST match in the nonredundant database for the 16S rRNA from the completed genome was to *Neisseria*. Using PubMLST, the strain was identified to be Neisseria mucosa, which was confirmed through an average nucleotide identity (ANI) calculation (http://enve-omics.ce.gatech.edu/ani) (8), with an ANI of 96.62% with respect to N. mucosa ATCC 19696. Only 17 genes had no homology to those in other N. mucosa strains (ATCC 19696, DSM 17713, FDAARGOS_260, FDAARGOS_758, and NS20201025) (BLASTN, E value of <1 × *e*^−10^), with one being highly homologous to a gene encoding a putative protein of Idiomarina loihiensis (BLASTN with the nucleotide collection database, E value of 9 × *e*^−20^).

N. mucosa was isolated from an aggressive periodontal lesion and identified as a major periodontal bacterial species. Seventeen unique and unknown genes were detected in strain HSUH001. It is necessary to investigate the functions of these unknown genes, especially in pathogenicity in periodontal disease, in the future. The genomic information for HSUH001 will provide useful insight for the elucidation of bacterial virulence in the future.

### Data availability.

This whole-genome shotgun sequencing project has been deposited in GenBank under the accession number JAFBIM000000000. The associated BioSample and BioProject accession numbers are SAMN16948501 and PRJNA681287, respectively. Raw sequencing reads are available under the SRA accession number SRR13810304.

## References

[1] Socransky SS, Haffajee AD. 2002. Dental biofilms: difficult therapeutic targets. Periodontology 2000 28:12–55. doi:10.1034/j.1600-0757.2002.280102.x. 12013340

[2] Kononen E, Muller HP. 2014. Microbiology of aggressive periodontitis. Periodontology 2000 65:46–78. doi:10.1111/prd.12016. 24738586

[3] Slots J. 1982. Selective medium for isolation of *Actinobacillus actinomycetemcomitans*. J Clin Microbiol 15:606–609. doi:10.1128/JCM.15.4.606-609.1982. 7068837 PMC272154

[4] Holm A, Rabe P, Kalfas S, Edwardsson S. 1987. Improved selective culture media for *Actinobacillus actinomycetemcomitans* and *Haemophilus aphrophilus*. J Clin Microbiol 25:1985–1988. doi:10.1128/JCM.25.10.1985-1988.1987. 3667919 PMC269382

[5] Nagasawa T, Shimizu S, Kato S, Nakatsuka Y, Kado T, Hidaka T, Shirai K, Mori M, Furuichi Y. 2014. Host-microbial co-evolution in periodontitis associated with *Aggregatibacter actinomycetemcomitans* infection. J Oral Biosci 56:11–17. doi:10.1016/j.job.2013.10.002.

[6] Kajitani R, Yoshimura D, Ogura Y, Gotoh Y, Hayashi T, Itoh T. 2020. Platanus_B: an accurate de novo assembler for bacterial genomes using an iterative error-removal process. DNA Res 27:dsaa014. doi:10.1093/dnares/dsaa014. 32658266 PMC7433917

[7] Seemann T. 2014. Prokka: rapid prokaryotic genome annotation. Bioinformatics 30:2068–2069. doi:10.1093/bioinformatics/btu153. 24642063

[8] Goris J, Konstantinidis KT, Klappenbach JA, Coenye T, Vandamme P, Tiedje JM. 2007. DNA-DNA hybridization values and their relationship to whole-genome sequence similarities. Int J Syst Evol Microbiol 57:81–91. doi:10.1099/ijs.0.64483-0. 17220447

